# Screening for Coding Variants in *FTO* and *SH2B1* Genes in Chinese Patients with Obesity

**DOI:** 10.1371/journal.pone.0067039

**Published:** 2013-06-25

**Authors:** Zhaojing Zheng, Li Hong, Xiaodong Huang, Peirong Yang, Juan Li, Yu Ding, Ru-en Yao, Juan Geng, Yongnian Shen, Yiping Shen, Qihua Fu, Yongguo Yu

**Affiliations:** 1 Department of Laboratory Medicine, Shanghai Children’s Medical Center, Shanghai Jiaotong University School of Medicine, Shanghai, P.R. China; 2 Department of Nutrition, Shanghai Children’s Medical Center, Shanghai Jiaotong University School of Medicine, Shanghai, P.R. China; 3 Department of Internal Medicine, Shanghai Children’s Medical Center, Shanghai Jiaotong University School of Medicine, Shanghai, P.R. China; 4 Department of Laboratory Medicine, Children’s Hospital Boston, Boston, Massachusetts, United States of America; Gentofte University Hospital, Denmark

## Abstract

**Objective:**

To investigate potential functional variants in *FTO* and *SH2B1* genes among Chinese children with obesity.

**Methods:**

Sanger sequencing of PCR products of all *FTO* and *SH2B1* exons and their flanking regions were performed in 338 Chinese Han children with obesity and 221 age- and sex-matched lean controls.

**Results:**

A total of seven and five rare non-synonymous variants were identified in *FTO* and *SH2B1,* respectively. The overall frequencies of *FTO* and *SH2B1* rare non-synonymous variants were similar in obese and lean children (2.37% and 0.90% vs. 1.81% and 1.36%, *P*>0.05). However, four out of the seven variants in *FTO* were novel and all were unique to obese children (p>0.05). None of the novel variants was consistently being predicted to be deleterious. Four out of five variants in *SH2B1* were novel and one was unique to obese children (p>0.05). One variant (L293R) that was consistently being predicted as deleterious in *SH2B1* gene was unique to lean control. While rare missense mutations were more frequently detected in girls from obesity as well as lean control than boys, the difference was not statistically significant. In addition, it's shown that the prevalence of rare missense mutations of *FTO* as well as *SH2B1* was similar across different ethnic groups.

**Conclusion:**

The rare missense mutations of *FTO* and *SH2B1* did not confer risks of obesity in Chinese Han children in our cohort.

## Introduction

Obesity is a major public health problem leading to increased mortality and morbidity [1], and obesity in childhood is strongly associated with increased incidence of premature death [2], [3].The prevalence of overweight and obesity in children has been increasing in the world [6], as well as in Asia [4], [5]. In Beijing and Shanghai, the prevalence of obesity increased by more than 3-fold from 1985 to 2000, up to 27% in boys and 16% in girls [7].The increasing prevalence of obesity is contributed by the excessive calorie intake and diminished physical activity in the modern environment. However, considerable evidence from familial segregation and twin studies suggest a significant genetic contribution to adiposity [8], [9]. Genetic factors are believed to modulate the impact of the affluent environment on each individual.

Genome-wide association studies (GWAS) have revealed that the single nucleotide polymorphisms (SNPs) in the first intron of fat mass- and obesity-associated gene (*FTO*) are significantly associated with BMI and increased risk for obesity [10], [11] and it is the strongest adiposity locus identified so far [12], [13].

SNPs in human SH2B adapter protein 1 gene (*SH2B1*) are also been shown to be associated with leptin resistance and obesity[14]–[17]. In addition, chromosomal deletions that eliminate the *SH2B1* gene are also associated with severe obesity and insulin resistance in human [18], [19].Genetic deletion of *SH2B1* results in severe leptin resistance, insulin resistance, obesity, and type 2 diabetes in mice [20], [21],and neuron-specific restoration of *SH2B1* reverses the obesity and type 2 diabetes phenotypes in *SH2B1* null mice [22]. Collectively, these findings strongly suggest that the *SH2B1* gene has a conserved role in the control of both body weight and metabolism homeostasis in rodents and humans.

In an effort to identifying potential functional variants in *FTO* gene, Meyre, et al [23] sequenced the coding regions of the gene in 1433 severe obese and 1433 lean Caucasian individuals. As a result, almost an equal number of non-synonymous variants were detected in obese and lean individuals, with more unique variants to the lean. Enzymatic activity analysis further suggested that loss-of-function variants are compatible with lean and did not impose increased risk for obesity. By deep resequencing using next generation sequencing (NGS) technology in 524 severely obese and 527 lean Swedish children, Almén et al, showed little evidence of functional variants in coding region of the *FTO* gene. They concluded that the first intron is the only region within the *FTO* gene associated with obesity [24].Similarly, Bochukova et al. sequenced the *SH2B1* gene in 500 Caucasian patients from the Genetics of Obesity Study (GOOS) but did not identify any coding or splice site mutations [18].Since all previous studies were undertaken mainly in Caucasian population, we aim to investigate the prevalence of rare missense mutations of *FTO* and *SH2B1* in Chinese children with early-onset obesity.

## Materials and Methods

### Ethics Statement

The study was approved by the Ethics Committee of Shanghai Children’s Medical Center, Shanghai Jiao Tong University School of Medicine. Informed written consents were obtained from all children's parents or guardians before blood sampling and DNA analyses.

### Subjects

Three hundred and thirty-eight Chinese children with BMI >97% percentile were included in this study. A total of 221 lean children with <50th percentile adjusted for sex and age were selected as control from Shanghai Children’s Sleep Project [25]. All study subjects were Han Chinese, and the anthropological characteristics of obese and lean children were summarized in Table 1.

**Table 1:** The anthropological characteristics of children with early-onset obesity and lean control.

	Obesity	Control	p value
N (boys/girls)	338(202/136)	221(127/94)	>0.05
Age	9.62±2.75	9.97±2.67	>0.05
BMI Z-score	3.38±1.45	−0.41±0.42	<0.01

Data are means±SD unless otherwise indicated.

### Sanger Sequencing

We screened the exonic and flanking regions of the *FTO* and *SH2B1* by Sanger sequencing. PCR primers (Table S1 & S2) were designed with Primer3 tool (http: //frodo.wi.mit.edu/primer3/) to contain exonic sequences and their flanking regions of 20 bp. PCR amplifications were inspected for single band of expected sizes on agarose gels before purification with Agencourt AMPure on Biomek NX (Beckman Coulter, USA). Sequencing was achieved using the automated ABI Prism 3730xlDNA Sequencer in combination with the Big Dye Terminator Cycle Sequencing Ready Reaction Kit 3.1 (Applied Biosystems, USA), and purification of sequencing reaction was performed with Agencourt CleanSEQ on Biomek NX(Beckman Coulter, USA). Sequences were assembled and analyzed with MutationSurveyor software (SoftGenetics, USA).

### Assessment of Functional Impacts of Non-synonymous Mutations

We assessed the functional impacts of *FTO* and *SH2B1* non-synonymous mutations by three software, i.e., Protein Variation Effect Analyzer (PROVEAN), Sorts Intolerant From Tolerant (SIFT) and Polymorphism Phenotyping v2 (Polyphen-2). The PROVEAN cutoff value for deleterious was set at −2.5 as instructed [26]. A default setup was used for SIFT and Polyphen-2 functional prediction.

### Statistics

The prevalence of *FTO* and *SH2B1* rare missense mutations between obese and lean subjects and between our cohort and literature data or public database were compared with Fisher's exact test or Chi-square test. Student's *t*-test and Mann-Whitney test were used to compare the means of age and BMI Z-score between obese and lean children, respectively. Statistics analysis was performed with SPSS17.0 software. A *P* value of <0.05 was considered significant throughout this study.

## Results

### Prevalence of *FTO* Non-synonymous Mutations

A total of seven rare (frequency <1% in the present cohort) non-synonymous variants *FTO* were identified in obese and lean children (Table 2). Among them, 4 of the 7 rare missense mutations were novel (not previously reported in 1000 genome, NHLBI GO Exome Sequencing Project (ESP), and the Human Gene Mutation Database (HGMD)). The prevalence of rare non-synonymous mutations in obese children was similar to that in lean control (2.37% vs. 1.81%, *P*>0.05).

**Table 2:** Prevalence of rare non-synonymous mutations in *FTO* in obese and lean children.

Variants	Obesity(n = 338)	Control(n = 221)
L91P	1	0
E129K	1	0
D144N	3	2
Y185C	1	0
H290R	1	0
D348N	0	2
S482L	1	0
Prevalence of mutation carriers	2.37%	1.81%
*P* value	>0.05	

Rare non-synonymous mutations denote variations with frequency<0.01.

We found that a subset of the rare non-synonymous mutations was unique to each group. Five (L91P, E129K, Y185C, H290R, S482L) variants were identified uniquely in the obese children, whereas only one (D348N) were identified uniquely in the lean control. D144N was present in both obese and lean children. All rare missense mutations were identified in heterozygous pattern. Thus, there was no obvious enrichment of non-synonymous mutations in lean or obese individuals.

Interestingly, we observed a higher frequency of *FTO* rare non-synonymous mutations in girls of obesity as well as of lean control in our cohort. However, the difference didn't reach a statistical significance (Table 3).

**Table 3:** Difference on prevalence of *FTO* rare non-synonymous mutation between boys and girls with obesity.

	Boys	Girls	*P* value
Obesity	1.48%(3/202)	3.68%(5/136)	>0.05
Control	0.79%(1/127)	3.19%(3/94)	>0.05

### Prevalence of *SH2B1* Non-synonymous Mutations

A total of 5 rare non-synonymous variants were identified in *SH2B1* in this cohort (Table 4). The prevalence of rare missense mutations in obese and lean children were 0.90% and 1.36%, respectively (*P*>0.05). Two (V209I and M465T) non-synonymous mutations were identified uniquely in obese children, whereas 3 (G131S, L293R, and W649G) were identified uniquely in lean control. One rare mutation, M465T, was identified in two obese children. All mutations were present in heterozygous pattern, and no nonsense variants were reported in the studied populations.

**Table 4:** Prevalence of *SH2B1* rare non-synonymous mutations in obese and lean children.

Variations	Obesity(n = 334)	Control(n = 221)
G131S	0	1
L293R	0	1
W649G	0	1
V209I	1	0
M465T	2	0
Prevalence of mutation carriers	0.90%	1.36%
*P* value	>0.05	

### Prevalence of *FTO* and *SH2B1* Rare Non-synonymous Mutations in Multiple Ethnicities

To explore the possible difference on the prevalence of *FTO* and *SH2B1* rare non-synonymous mutations in different ethnicities, we compared our results with Meyre's [23] data of French and English children with obesity and coding variants data from Exome Variant Server, NHLBI GO Exome Sequencing Project (ESP), Seattle, WA (URL: http: //evs.gs.washington.edu/EVS/). The results showed that the prevalence of rare non-synonymous mutations of *FTO* in Chinese obese children (2.37%, 8/338) was comparable to the results of Meyer's study [23] (1.32%, 9/680), European American (2.23%, 96/4300), and African American (1.64%, 36/2198) from ESP. Similarly, the prevalence of *SH2B1* rare missense mutations didn't differ significantly between Chinese obese children (0.90%, 3/334) and European American (1.79%, 77/4300) as well as African American (2.50%, 55/2197) from ESP, though there's a marginal difference between the latter two populations (*P* = 0.054) (Table 5).

**Table 5:** Prevalence of *FTO* and *SH2B1* rare non-synonymous mutations in different populations.

		Ourcohort	Meyre’s[^22^]	EA_ESP	AA_ESP
	Population	CHC	FC/EC	EA	AA
*FTO*	*N*	338	680	4300	2198
	Rare missense	8	9	96	36
	Prevalence (%)	2.37	1.32	2.23	1.64
*SH2B1*	*N*	334	−	4300	2197
	Rare missense	3	−	77	55
	Prevalence (%)	0.90	−	1.79	2.50

ESP: NHLBI Exome Sequencing Project; CHC: Chinese Han children; FC/EC: French children/English children from Meyre's cohort [22]; EA: European American; AA: African American.

### Functional Impacts of *FTO* and *SH2B1* Non-synonymous Mutations

Among *FTO* rare non-synonymous mutations, L91P and S482L were predicted deleterious, and all *SH2B1* non-synonymous mutations but L293R were predicted neutral with the three tool software (Table 6). The functional impacts of non-synonymous mutations of *FTO* and *SH2B1* did not differ between obese and lean children (*P*>0.05).

**Table 6:** Functional impacts of *FTO* and *SH2B1* rare non-synonymous mutations.

Gene	Variants	PROVEAN	SIFT	Polyphen-2
*FTO*	L91P	Deleterious	Tolerated	Probably damaging
	E129K	Neutral	Tolerated	Benign
	D144N	Neutral	Tolerated	Possibly damaging
	Y185C	Neutral	Tolerated	Benign
	H290R	Neutral	Tolerated	Benign
	D348N	Neutral	Tolerated	Benign
	S482L	Neutral	Damaging	Possibly damaging
*SH2B1*	G131S	Neutral	Tolerated	Benign
	L293R	Deleterious	Damaging	Probably damaging
	V209I	Neutral	Tolerated	Benign
	M465T	Neutral	Tolerated	Benign
	W649G	Neutral	Damaging	Benign

PROVEAN: Protein Variation Effect Analyzer; SIFT: Sorts Intolerant From Tolerant; Polyphen-2: Polymorphism Phenotyping v2.

## Discussion

Recent findings in *Fto*-null mice support the notion that Fto itself has an important influence on energy balance. *Fto*-null mice are small, are lean, have an increased metabolic rate, and are hyperphagic, whereas *Fto* ^−/−^ mice are resistant to diet-induced obesity [27]. In contrary, over-expression in mice resulted in increased energy intake and increased adiposity in a dose-dependent manner [28], in agreement with the *FTO* dose-dependent differences in adiposity seen in humans [29]. Taken together, these data suggest that increased *FTO* expression results in increased food intake, leading to increased adiposity. Thus, a gain-of-function effect is suggested for the pathogenic role of FTO in human obesity.

Given that information, it is reasonable to speculate that function-impairing mutations in *FTO* might be more common in lean rather than obese subjects.

However, our results and data from obese children of Caucasians by Meyre, et al [23] and of African Ancestry by Deliard et al [30] clearly demonstrate that the rare non-synonymous mutations are not enriched in lean control.

**Figure 1:**
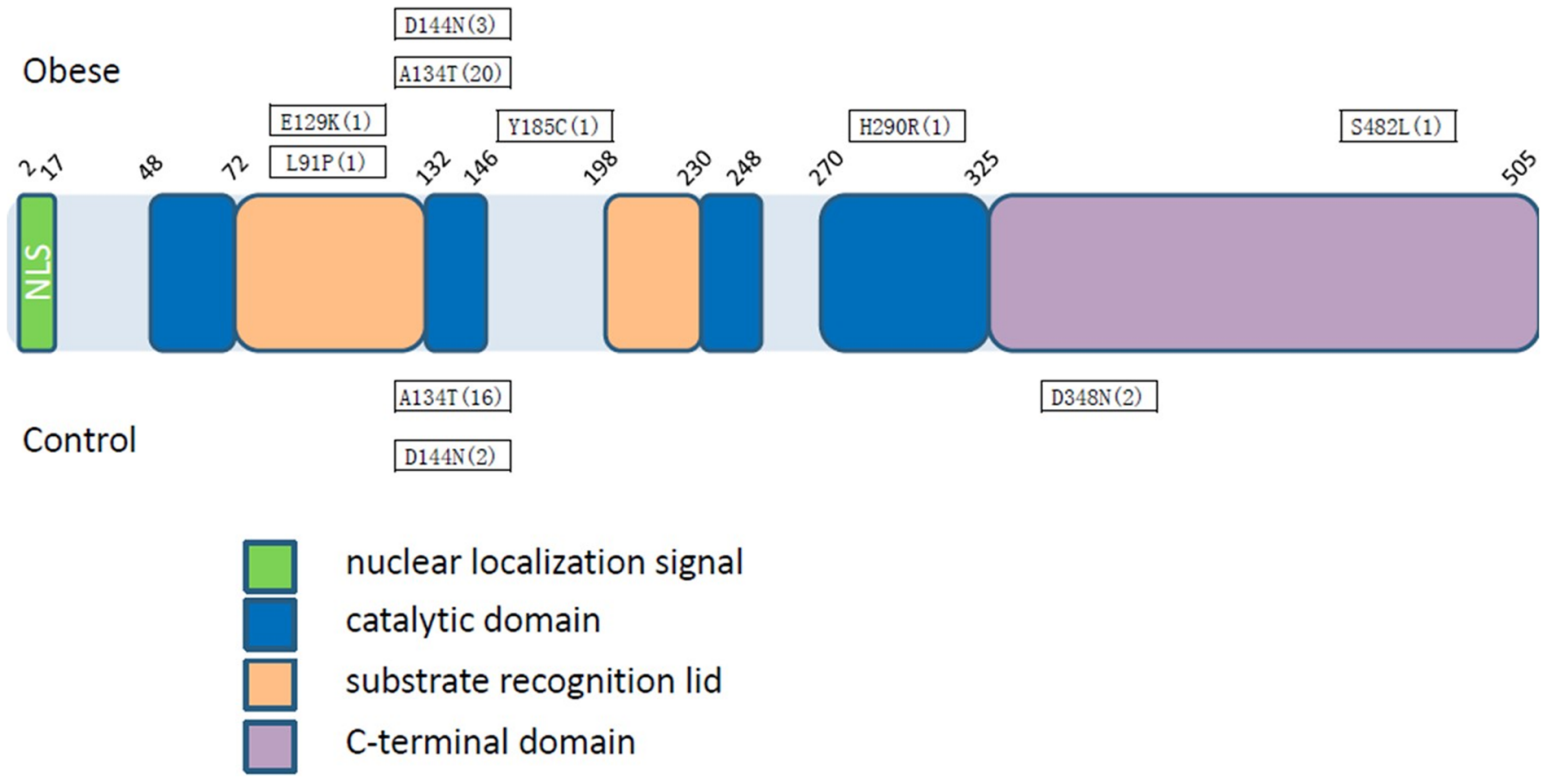
Distribution of rare non-synonymous variants along FTO functional domains. FTO protein is consisted of a N-terminal domain (NTD, residue 32–326) and a C-terminal domain (CTD, residue 327–505). Different non-synonymous mutations with frequency in parenthesis were presented both for obese children and control population.

FTO protein is an AlkB-like DNA/RNA demethylase with a strong preference for 3-methylthymidine (3-meT) in single-stranded DNA or 3-methyluracil (3-meU) in single-stranded RNA[30]–[34]. More recently, Jia et al [35] demonstrated that FTO could efficiently demethylate N6-methyladenosine (m^6^A) at neutral pH *in vitro* and that the amount of m^6^A in cellular mRNA is affected by the oxidation activity of FTO *in vivo*, concluding that m^6^A acts as the natural substrate for FTO in physiological condition. FTO is consisted of an amino-terminal AlkB-like domain (NTD) and a carboxyl-terminal domain (CTD) with a novel fold. The interaction between these two domains is required for FTO catalytic activity [36].FTO possesses an extra loop (residues 213–224) that it has an important role in FTO selection against double-stranded nucleic acids [36].H231, D233, H307, and residues within the extra loop are highly conserved among FTO proteins from different species. Biochemical assay shows that mutations at F114, C392, R96, and E234 abolish or greatly reduce the catalytic activity of FTO [36]. Boissel, et al [31] found a consanguineous Israeli-Arab family in which nine siblings were homozygous for the R316Q mutation in *FTO,* which resulted in a diminished catalytic activity. All homozygous carriers were severely growth retarded, had multiple congenital malformations, and died in infancy, and heterozygous parents of these children had no obesity or overweight phenotype. Similarly, R322 is also essential for FTO catalytic activity, and heterozygous mutations that severely impaired enzymatic activity of FTO were found in both lean and obese individuals with no other obvious major clinical phenotypes [23]. Furthermore, the R316Q mutation abolished 80% of the wild-type activity toward m^6^A demethylation in vitro, while H231A D233A and R316Q R322Q double mutant FTO proteins completely lost m^6^A-demethylation activity, suggesting that m^6^A in nuclear RNA is the physiological substrate of FTO and that the function of FTO likely affects the processing of pre-mRNA, other nuclear RNAs, or both [35]. Two non-synonymous mutations (L91P and S482L), that are solely detected in obese children, are within the so-called substrate recognition lid and CTD of FTO protein, respectively (Figure 1.), and predicted to compromise FTO catalytic activity. Meyer et al [23] also tested the enzymatic activity of several other variants representing the different regions of FTO (P5L,V94I, I148R, M223V, E234D, A241T, A405V, I492V, and V493F), and found none of these variants, all of which are located in less conserved positions, had any significant impact on enzymatic activity. The observed activity of FTO is exceedingly low compared to those of the other AlkB-family proteins [37], it's reasonable to speculate that with M^6^A as a substrate, the functional impacts of the mutant FTO proteins including L91P and S482L identified in this study could be more accurately assessed.

As heterozygous deletion of *SH2B1* in mice leads to obesity on a high-fat diet [22], Bochukova et al [18] suggested that *SH2B1* gene haploinsufficiency may be a plausible mechanism underlying the phenotype seen in humans. However, Bochukova et al. sequenced the *SH2B1* gene in 500 patients from the Genetics of Obesity Study (GOOS) but did not identify any coding or splice site mutations [18]. Alike to *FTO*, a similar prevalence of rare missense mutations of *SH2B1* is revealed in Chinese obese children and lean control. Among non-synonymous mutations of *SH2B1* detected in this cohort, only one (L293R) is predicted deleterious to protein function by tool software. L293 locates in the pleckstrin homology (PH) domain (residues 249–378) of SH2B1 protein, which is thought to mediate binding to inactive JAK2, whereas the SH2 domain (residues 521–625) near the C terminus is critical to binding to active, phosphorylated JAK2 [38]. Though *SH2B1* point mutations may significantly impair the growth hormone (GH)/nerve growth factor (NGF)-mediated signaling in vitro, all mutants except F344LfsX20 did not impair leptin signaling [39]. It's plausible to speculate that loss-of-function mutation of *SH2B1* may not significantly involved in pathogenesis of Chinese children obesity.

In conclusion, rare non-synonymous mutations of *FTO* and *SH2B1* are equally detected in obese and lean Chinese children. The rare missense mutations of *FTO* and *SH2B1* do not confer risks of obesity in Chinese Han children in our cohort. Novel and non-deleterious missense variants detected in *FTO* unique to obese individuals are of significant interest to reveal their potential functional impact on FTO protein.

## Supporting Information

Table S1 PCR primers for amplification of *FTO* coding regions.
(DOCX)

Table S2 PCR primers for amplification of *SH2B1* coding regions.
(DOCX)
