# Supplementary material for: Screening for Coding Variants in FTO and SH2B1 Genes in Chinese Patients with Obesity
Source: PLoS One. 2013 Jun 25;8(6):e67039. doi: 10.1371/journal.pone.0067039 (PMC3692548; doi:10.1371/journal.pone.0067039)
Supplement: Table S1 — PCR primers for amplification of FTO coding regions. (DOCX) [file pone.0067039.s001.docx]

Supplementary Table S1. PCR primers for amplification of *FTO* coding regions.

| Exon | Forward | Reverse | Size(bp) |
| --- | --- | --- | --- |
| #1 | CTCCTGTGCTAAATCCCGTG | AAGGCCCTGTCATCCCTTAG | 356 |
| #2 | TCAAAGTTGGCTAAAATTTGTTTG | AAGGAAGTGTGGAATGTCCG | 422 |
| #3 | ATAGCCACCAGGTAGTCCCC | GCATGTACACACGCAAGCAC | 868 |
| #4 | ACACCTGGCCTATTTAAGG | CAACAAGAGTGAAGCTCC | 560 |
| #5 | AGCATCCCCTTTCACTCTTC | ACTTGTCAACGGCATTTTCC | 751 |
| #6 | TGAATTCACAGCCAGGGAC | CACAATTCTTGAAAGTCTTGCC | 571 |
| #7 | TGCCAGCTTACACTGGGAAC | TCCTGGCTATACCCATCACC | 426 |
| #8 | GGCCATCATTTACTGCATTG | ATTAATGTAGGTGCCGTGGG | 441 |
| #9 | CCTCCCGTGGATTAATTTCC | CAACGTTGGAGGAGAAAAGC | 250 |
