# Supplementary material for: Screening for Coding Variants in FTO and SH2B1 Genes in Chinese Patients with Obesity
Source: PLoS One. 2013 Jun 25;8(6):e67039. doi: 10.1371/journal.pone.0067039 (PMC3692548; doi:10.1371/journal.pone.0067039)
Supplement: Table S2 — PCR primers for amplification of SH2B1 coding regions. (DOCX) [file pone.0067039.s002.docx]

Supplementary Table S2. PCR primers for amplification of *SH2B1* coding regions

| Exon | Forward | Reverse | Size(bp) |
| --- | --- | --- | --- |
| #1 | AGTAGGGTCGGACGTCTCTG | CCAACAAAAAGTGAGCGACA | 1240 |
| #2 | GGCAGGACTGAGAAAGCAGT | TTTCCCTGTCTTCCGCTATC | 406 |
| #3-4 | ACCCGGCCTCCAGAGAG | TAGGGCATCTGGAAAACAGG | 627 |
| #5-7 | GGTAAAAGCATCAGGGGTCA | AGGCACAACAGCCTCCTCTA | 1224 |
| #8-9 | ATTCCATCGGATCCTCTGTTC | CTATGGCCTCTTCCAATTCAA | 722 |
